# Comparison of Unemployment-Related Health Insurance Coverage Changes in Medicaid Expansion vs Nonexpansion States During the COVID-19 Pandemic

**DOI:** 10.1001/jamahealthforum.2022.1632

**Published:** 2022-06-17

**Authors:** Joseph Benitez

**Affiliations:** 1 Department of Health Management & Policy, College of Public Health, University of Kentucky, Lexington

## Abstract

**Importance:**

The COVID-19 pandemic has been associated with increased unemployment rates and long periods when individuals were without health insurance. Little is known about how Medicaid expansion facilitates Medicaid enrollment as a buffer to coverage loss owing to unemployment.

**Objective:**

To compare changes in health insurance coverage status associated with pandemic-related unemployment among previously employed adults in states that have vs have not expanded Medicaid eligibility.

**Design, Setting, and Participants:**

This cohort study included US adults aged 27 to 64 years who were employed at baseline in the 2020 to 2021 Current Population Survey’s Annual Social and Economic Supplement, which included calendar years 2019 to 2020 (32 462 person-years). Data analyses were conducted between November 2021 and April 2022.

**Exposures:**

Job loss (ie, new unemployment) experienced during 2020.

**Main Outcomes and Measures:**

Primary outcomes were coverage status (ie, uninsured status) and source of coverage (ie, employer sponsored, marketplace, and Medicaid). Using 2-way person-by-year fixed-effects regression models, changes in coverage status associated with unemployment in states that expanded Medicaid were compared with states that did not expand Medicaid. Additional analyses were performed based on prepandemic coverage status.

**Results:**

The cohort included 16 231 adults (mean age, 46.8 [95% CI, 46.6-47.0] years; 51.6% women). New unemployment was associated with an increase of 2.9 (95% CI, 1.1-4.6) percentage points (*P* = .002) in the proportion of uninsured adults in Medicaid expansion states and an increase of 10.7 (95% CI, 2.4-18.9) percentage points (*P* = .01) in nonexpansion states. Workers were 5.4 (95% CI, 1.9-8.9) percentage points (*P* = .003) more likely to enroll in Medicaid after a job loss if they lived in a Medicaid expansion state compared with workers experiencing job loss in nonexpansion states.

**Conclusions and Relevance:**

In this cohort study of US adults, unemployment-related Medicaid enrollment was more frequent in Medicaid expansion states during the COVID-19 pandemic. Medicaid expansion led to a smaller increase in uninsured adults because those who lost private insurance coverage (eg, employer sponsored) appeared more able to transition to Medicaid after job loss.

## Key Points

**Question:**

How did health insurance coverage status change in states with Medicaid eligibility expansion vs nonexpansion for US adults who experienced unemployment owing to the COVID-19 pandemic?

**Findings:**

In this cohort study of 16 231 adult workers employed at baseline and followed up from 2019 through 2020, increases in Medicaid enrollment associated with job loss were more frequent in states that expanded Medicaid eligibility vs nonexpansion states. The risk of losing insurance owing to job loss in expansion states was lower than the risk in nonexpansion states.

**Meaning:**

The findings of this study suggest that expanded Medicaid eligibility may have allowed households affected by job loss during the COVID-19 pandemic to access Medicaid as a buffer against insurance loss.

## Teaser

This cohort study compares changes in health insurance coverage for previously employed US adults in states with and without Medicaid expansion during the COVID-19 pandemic.

## Introduction

The COVID-19 crisis—the pandemic itself and the accompanying recession—is the first major economic downturn in the US since the 2007 to 2009 Great Recession.^1^ Peak unemployment during the COVID-19 crisis reached 14.8% in April 2020, almost 4.3 times higher than the prepandemic rate of 3.5%. Many of the newly unemployed as well as those experiencing work instability likely experienced difficulty maintaining health insurance coverage—an immediate threat to maintaining access to needed health care.

More than 18 million people were considered at risk of losing employer-sponsored health insurance benefits during the pandemic, potentially increasing the national uninsured rate from 8.8% to as high as 14.5%.^2^ Recent reports suggest that many people adversely affected by the COVID-19 crisis will turn to Medicaid coverage, whereas others could maintain coverage through the nongroup marketplaces created by the Patient Protection and Affordable Care Act (ACA).^3^ Almost two-thirds of the US receives health coverage through employer-sponsored health insurance, suggesting that unanticipated periods of unemployment could mean stark declines not only in health coverage but also disruptions in normal access to needed health care.^4^ The extent that people experiencing job losses during the pandemic have transitioned to Medicaid or marketplace coverage is unclear.

Households adversely affected by unfavorable economic conditions increasingly enrolled in Medicaid when their states had more expansive eligibility pathways.^5^ Recent studies show that Medicaid enrollment during the COVID-19 crisis increased in states that expanded Medicaid.^6,7^ However, the extent that increased Medicaid enrollments were predicated by job loss is unknown.^7^ Whether Medicaid can function as a safety net depends largely on how easily workers can transition from private coverage to Medicaid after a job loss. This study assesses changes in health insurance coverage owing to job loss in states that expanded Medicaid eligibility under the ACA (ie, expansion states) compared with states that did not expand Medicaid (ie, nonexpansion states).

## Methods

This cohort study was deemed exempt from review by the University of Kentucky Institutional Review Board, and the informed consent requirement was waived because the data used for all analyses were publicly available and used for regular monitoring of the economic health of the US and individual states. The study followed the Strengthening the Reporting of Observational Studies in Epidemiology (STROBE) reporting guideline.

### Survey Sample and Outcomes

This cohort study used the longitudinal component of the 2020 to 2021 Current Population Survey’s Annual Social and Economic Supplement (CPS-ASEC; which included calendar years 2019-2020), a nationally representative, population-based survey used to monitor patterns in poverty, employment and labor market participation, and other key economic indicators. Data analyses were conducted between November 2021 and April 2022. We used the harmonized version of the survey developed by the Integrated Public Use Microdata Series team at the University of Minnesota.^8^

Sample households are included in the CPS-ASEC for up to 2 years. The analyses included a panel of US adults aged 27 to 64 years who indicated that they were employed for all of 2019—the start of their inclusion in the survey. The statistical unit of analysis was a person-year, and the study used a cohort of workers with data before (ie, 2019) and during (ie, 2020) the COVID-19 pandemic (32 462 person-years). The sample was limited by age to minimize confounding by the ACA’s dependent coverage mandate, which may facilitate another safety net for young adults (up to age 26 years) whose coverage status might have been altered by job loss (eFigure 1 in the Supplement shows sample construction). All demographic data, including race and ethnicity, in the survey were collected via interview based on self-report from the household’s respondent. Racial and ethnic categories included in the study were Hispanic (any race), non-Hispanic Black, and non-Hispanic White. Other racial and ethnic subgroups were included; however, these subgroups were categorized as non-Hispanic other because of the limited sample sizes in these subgroups.

The CPS-ASEC has rich data on the sources of health insurance coverage held by the sample person. Key outcomes are changes in the proportion of the sample with (1) employer-sponsored health insurance, (2) nongroup marketplace health insurance (eg, ACA exchanges), (3) Medicaid (any enrollment, enrolled part of the year, or enrolled all year), and (4) no health insurance of any kind. The CPS-ASEC is largely a look-back survey; therefore, respondents indicated the sources of coverage they had in the past year (ie, at any point for any duration).

### Statistical Analysis

**Figure 1.**
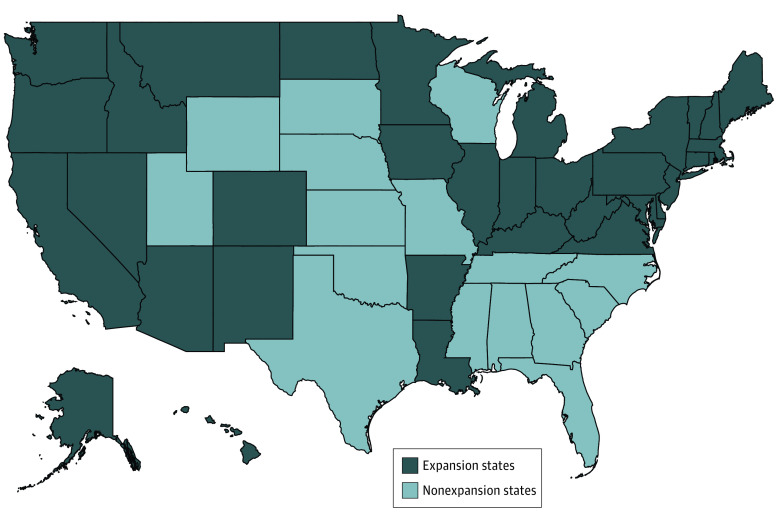
State Medicaid Expansion Status as of January 2020

To assess whether Medicaid expansion facilitated Medicaid enrollment among the newly unemployed during the COVID-19 pandemic, a 2-way person-by-year, fixed-effect regression model was used to capture coverage changes among this group. The key sources of exposure were (1) whether the person became unemployed during the study period (ie, were newly unemployed for 1 or more weeks during 2020) and (2) whether they lived in an expansion state (n = 36) or a nonexpansion state (n = 15) as of January 2020 (nonexpansion states included Alabama, Florida, Georgia, Kansas, Mississippi, Missouri, Nebraska, North Carolina, Oklahoma, South Carolina, South Dakota, Tennessee, Texas, Wisconsin, and Wyoming; Figure 1). A more detailed description of the methods is provided in the eMethods in the Supplement.

Key analyses were limited to persons who indicated that they were working for all of 2019 (ie, reported no periods of unemployment). We performed additional subanalyses restricting the sample to working adults (1) with private health insurance coverage (ie, employer-sponsored health insurance or nongroup) in 2019 and those (2) with private coverage who were not enrolled previously in Medicaid. The subgroup analyses allowed us to focus on workers whose coverage status could be sensitive to employment status, facilitating better generalizability to the group in which Medicaid could function as a safety net. With respect to differences across expansion and nonexpansion states, these additional analyses provided a means to capture (1) transitions from private insurance to Medicaid stemming from job loss and (2) new enrollment in Medicaid associated with job loss during the study period.

In addition to time-varying controls, year effects, and person fixed effects, we also included linear time patterns specific to the metropolitan statistical area where the sample person lived to control for variation in the timing and intensity of local efforts (eg, stay-at-home orders) to curb COVID-19 infections.^9,10,11,12,13^ However, these local mandates or other actions may have also exacerbated the risk of COVID-19–related unemployment.^9^

Becoming unemployed during the pandemic was nonrandom, and workers unable to work remotely were at the highest risk of becoming unemployed when states implemented stay-at-home orders to curb COVID-19 infections.^9,10,11^ Changes in coverage and employment status could be owing to the onset of locally implemented stay-at-home or shelter-in-place orders.^9^ Although there were variations between states in implementing stay-at-home orders, there was also within-state variation across cities that may have adopted orders that were either implemented earlier or had a longer duration than their state required.^12,13^ Being unable to observe the precise timing, duration, or intensity of any local mandates to curb COVID-19 infections, the regressions included interactions between metropolitan statistical area and time to control for area-specific differences in changes of levels of the outcomes across localities during the study period.

To affirm that the key findings are unique to the COVID-19 pandemic, we added a supplementary analysis by applying this approach to the 2018 to 2019 CPS-ASEC, which spanned calendar years 2017 to 2018. All statistics presented in the study used the sample weights to account for the survey’s complex sampling design. All regressions were estimated using linear probability models so that the regression coefficients (ie, β_1_ and β_2_ in the eMethods in the Supplement) for unemployment and unemployment’s interaction with expansion status were interpretable as policy parameters. Although the analyses included repeated observations of individuals during the study period, SEs robust to clustering at the state level—the source of the key policy variation—were used.^14^ Using SEs clustered at the state level rather than the individual level was a conservative approach because it accounted for the possible serial correlation of observing states over time. All statistical analyses were conducted from November 2021 to April 2022 using Stata, version 15.1 (StataCorp LLC). The threshold for statistical significance in the multivariable regression models was a 2-tailed *P* < .05.

## Results

The study cohort included 16 231 US adults (mean age, 46.8 [95% CI, 46.6-47.0] years) who were continuously employed through 2019. Among expansion states, the mean age was 46.7 (95% CI, 46.4-46.9) years, with 51.2% women and 48.8% men. In nonexpansion states, the mean age was 47.0 years (95% CI, 46.6-47.3), with 52.5% women and 47.5% men. During the pandemic, 8.2% (95% CI, 7.6%-8.8%) of the sample in expansion states and 6.3% (95% CI, 5.6%-7.1%) of the sample in nonexpansion states were unemployed in 2020 (eTable 1 in the Supplement). Nonexpansion states, when compared with expansion states, had a larger proportion of Black residents (12.4% [95% CI, 11.5%-13.4%] vs 6.9% [95% CI, 6.4%-7.5%]), lower levels of educational attainment (bachelor’s degree or higher, 37.7% [95% CI, 36.2%-39.2%] vs 44.1% [95% CI, 43.0%-45.2%]), and a higher proportion of nonmetropolitan area residents (16.5% [95% CI, 15.5%-17.6%] vs 12.8% [95% CI, 12.1%-13.6%]). Nonexpansion states also had a smaller number of workers with private coverage at baseline than expansion states (76.1% [95% CI, 74.8%-77.4%] vs 79.1% [95% CI, 78.2%-80.0%]), but a comparatively smaller number would become unemployed in 2020 based on both the CPS-ASEC and the monthly CPS.

**Figure 2.**
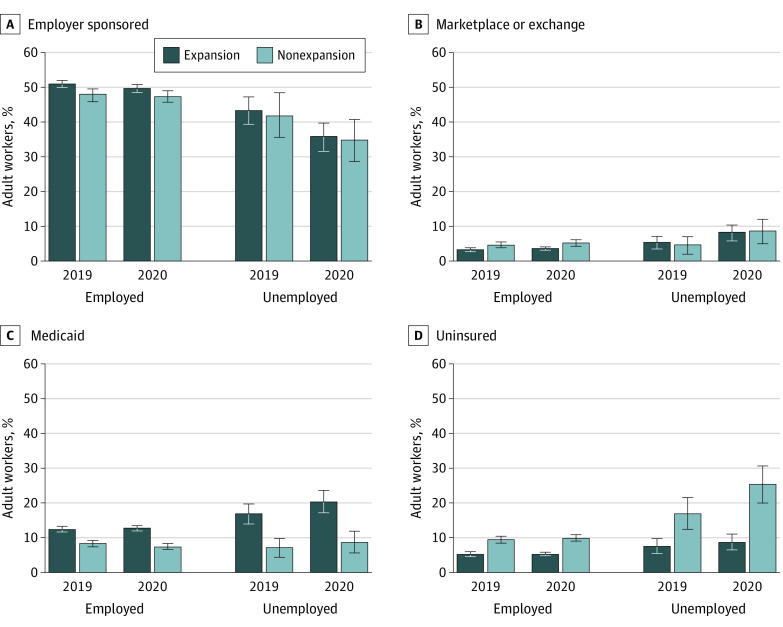
Health Insurance Coverage Levels by State Medicaid Expansion Status and 2020 Employment Status Samples were limited to adults who indicated that they were employed during all of 2019 (the base year of the analysis). Employed sample persons were those who remained employed in 2020; unemployed persons were those who became unemployed in 2020. State Medicaid expansion was based on whether states had expanded Medicaid as of January 2020. Nonexpansion states included Alabama, Florida, Georgia, Kansas, Mississippi, Missouri, Nebraska, North Carolina, Oklahoma, South Carolina, South Dakota, Tennessee, Texas, Wisconsin, and Wyoming. All statistics presented are weighted to reflect the complex sampling design of the survey. Data are from the 2021 Integrated Public Use Microdata Series team version of the Current Population Survey’s Annual Social and Economic Supplement. The error bars represent the 95% CIs.

Figure 2 presents the levels of different types of health insurance coverage in 2019 and 2020 in expansion states and nonexpansion states. The graph presents the 2 key subgroups of workers in the study: (1) workers who remained employed in 2020 and (2) workers who were unemployed for some duration during 2020. At baseline, workers who were unemployed had lower levels of employer-sponsored health insurance than workers who remained employed the entire year. Workers who were unemployed in 2020 were more likely to have nongroup (ie, marketplace or exchange) coverage in both expansion and nonexpansion states. Baseline Medicaid enrollment and the proportion of uninsured adults were also higher among adults who were unemployed.

There were relatively few changes in coverage among workers who remained employed in 2020, and this finding held true for expansion and nonexpansion states. Among workers who became unemployed, employer-sponsored health insurance coverage decreased from 43.3% in 2019 to 36.0% in 2020 among expansion states and 42.0% in 2019 to 34.7% in 2020 among nonexpansion states.

Medicaid enrollment among workers who remained employed was unchanged between 2019 and 2020. In expansion states, Medicaid enrollment among workers who became unemployed increased 3.6 percentage points in 2020 from 17.0% in 2019 to 20.6% in 2020. In nonexpansion states, Medicaid enrollment increased from 7.3% in 2019 to 8.9% in 2020. The proportion of newly unemployed workers who reported being uninsured in 2020 increased by 1.4 percentage points from a baseline of 7.5% in 2019 in expansion states, whereas the proportion of newly unemployed workers without health insurance increased by 8.4 percentage points in 2020 from 17.0% in 2019 in nonexpansion states.

### Regression Analyses

The Table contains results from the key regression analyses. In the full sample of workers employed at baseline (32 462 person-years), unemployment was associated with a net decrease of 3.0 (95% CI, –6.5 to 0.5) percentage points (*P* = .09) and a decrease of 7.1 (95% CI, –16.4 to 2.2) percentage points (*P* = .13) in private coverage in expansion and nonexpansion states, respectively. Unemployment was associated with comparable decreases in employer-sponsored health insurance in expansion and nonexpansion states. Medicaid enrollment associated with unemployment was higher in expansion states (3.7 percentage points; 95% CI, –0.1 to 7.5 percentage points; *P* = .06) than in nonexpansion states (0.9 percentage points; 95% CI, –2.4 to 4.2 percentage points; *P* = .58), and there were no statistically significant (4.1 percentage points; 95% CI, –5.8 to 13.9 percentage points; *P* = .41) differences in net coverage changes associated with job loss across expansion and nonexpansion states among the full sample.

**Table.** Changes in Coverage Associated With Unemployment Among Workers in Medicaid Expansion and Nonexpansion States^a^

Insurance status	Absolute change in expansion states		Absolute change in nonexpansion states^b^		Relative difference between expansion and nonexpansion states	
	Point estimate (95% CI)	*P* value	Point estimate (95% CI)	*P* value	Point estimate (95% CI)	*P* value
**Full sample (32 462 person-years)**						
Private coverage (any)	–3.0 (–6.5 to 0.5)	.09	–7.1 (–16.4 to 2.2)	.13	4.1 (–5.8 to 13.9)	.41
Employer-sponsored plan	–7.1 (–16.63 to 2.2)	.13	–3.0 (–6.5 to 0.5)	.09	–2.7 (–11.3 to 6.0)	.53
Marketplace or exchange	2.4 (0.3 to 4.5)	.03	2.2 (–0.8 to 5.1)	.14	0.2 (–3.4 to 3.9)	.90
Medicaid	3.7 (–0.1 to 7.5)	.06	0.9 (–2.4 to 4.2)	.58	2.8 (–2.2 to 7.8)	.27
Medicaid part of year	4.0 (2.2 to 5.7)	<.001	1.2 (–0.4 to 2.7)	.14	2.8 (0.4 to 5.1)	.02
Medicaid all year	–0.3 (–3.5 to 3.0)	.88	–0.3 (–2.9 to 2.4)	.85	0.006 (–4.1 to 4.1)	.99
Uninsured	1.6 (–0.9 to 4.1)	.20	7.8 (–0.9 to 16.5)	.08	–6.15 (–14.9 to 2.6)	.16
**Private coverage at baseline (25 086 person-years)**						
Private coverage (any)	–4.8 (–8.3 to –1.3)	.01	–11.4 (–21.0 to –1.9)	.02	6.6 (–3.2 to 16.4)	.18
Employer-sponsored plan	–9.3 (–12.9 to –5.8)	<.001	–5.2 (–16.0 to 5.5)	.33	–4.1 (–15.4 to 7.2)	.47
Marketplace or exchange	1.8 (–0.9 to 4.4)	.19	2.3 (–1.6 to 6.2)	.25	–0.5 (–5.2 to 4.3)	.83
Medicaid	6.1 (3.9 to 8.4)	<.001	0.7 (–1.9 to 3.3)	.58	5.4 (1.9 to 8.9)	.003
Medicaid part of year	3.9 (2.3 to 5.5)	<.001	0.8 (–0.4 to 1.9)	.19	3.1 (1.1 to 5.1)	.003
Medicaid all year	2.2 (0.06 to 4.4)	.04	–0.1 (–2.3 to 2.2)	.97	2.3 (–0.8 to 5.3)	.15
Uninsured	2.9 (1.1 to 4.6)	.002	10.7 (2.4 to 18.9)	.01	–7.8 (–16.0 to 0.4)	.06
**Private coverage or no previous Medicaid enrollment (24 542 person-years)**						
Private coverage (any)	–3.8 (–6.9 to –0.7)	.02	–11.8 (–20.9 to –2.7)	.01	8.0 (–1.3 to 17.2)	.09
Employer-sponsored plan	–8.6 (–12.1 to –5.0)	<.001	–6.5 (–17.9 to 4.9)	.26	–2.1 (–13.9 to 9.8)	.73
Marketplace or exchange	2.0 (–0.8 to 4.7)	.16	2.45 (–1.7 to 6.6)	.24	–0.5 (–5.5 to 4.6)	.85
Medicaid	5.1 (2.4 to 7.9)	<.001	0.7 (–1.9 to 3.3)	.58	4.4 (0.6 to 8.3)	.02
Medicaid part of year	3.1 (1.7 to 4.4)	<.001	0.2 (–0.9 to 1.3)	.73	2.9 (1.1 to 4.6)	.001
Medicaid all year	2.1 (–0.2 to 4.4)	.08	0.5 (–1.7 to 2.8)	.63	1.5 (–1.6 to 4.7)	.33
Uninsured	2.7 (1.0 to 4.3)	.002	11.1 (2.6 to 19.5)	.01	–8.4 (–16.8 to –0.005)	.05

^a^ Data are from the 2020 to 2021 Integrated Public Use Microdata Series team version of the Current Population Survey’s Annual Social and Economic Supplement. Results were obtained using linear person-by-year, fixed-effects regression models and were scaled by 100 to improve interpretability. All regressions used SEs robust to clustering at the state level. Regressions also included controls for marital status, education, sex, age, race and ethnicity, occupation and industry, and area-specific time patterns. All statistics presented are weighted to reflect the complex sampling design of the survey. Twenty-two sample persons (44 person-years) from the original sample were dropped because of singleton observations within the occupational fixed effects.

^b^ States that had not expanded Medicaid as of January 2020 were classified as nonexpansion states: Alabama, Florida, Georgia, Kansas, Mississippi, Missouri, Nebraska, North Carolina, Oklahoma, South Carolina, South Dakota, Tennessee, Texas, Wisconsin, and Wyoming.

Among workers with private coverage at baseline (25 086 person-years), job loss was associated with a decrease of 9.3 (95% CI, –12.9 to –5.8) percentage points (*P* < .001) in employer-sponsored health insurance in Medicaid expansion states and a decrease of 5.2 (95% CI, –16.0 to 5.5) percentage points (*P* = .33) in employer-sponsored health insurance in nonexpansion states. However, there was no statistically meaningful difference in the unemployment-related decrease in employer-sponsored health insurance enrollment across states. After a job loss among workers with baseline private coverage, Medicaid enrollment increased by 6.1 (95% CI, 3.9-8.4) percentage points (*P* < .001) in expansion states; Medicaid enrollment increased by 0.7 (95% CI, –1.9 to 3.3) percentage points (*P* = .58) among those experiencing job loss in nonexpansion states. Workers were 5.4 (95% CI, 1.9-8.9) percentage points (P = .003) more likely to enroll in Medicaid after a job loss if they lived in a Medicaid expansion state compared with workers experiencing job loss in nonexpansion states. Job loss in expansion states was associated with a net increase of 2.9 (95% CI, 1.1-4.6) percentage points (*P* = .002) in the proportion of uninsured adults. Job loss among this group of workers in nonexpansion states was associated with an increase in uninsured adults of 10.7 (95% CI, 2.4-18.9) percentage points (*P* = .01). The relative difference in the proportion of uninsured adults after job losses was –7.8 (95% CI, –16.0 to 0.4) percentage points (*P* = .06) larger among expansion states than among nonexpansion states.

Restricting the sample further to people with no previous Medicaid coverage (ie, workers who were not enrolled in Medicaid in 2019) (n = 24 542 person-years) produced results similar to those described previously. In expansion states, there was a net increase of 3.1 (95% CI, 1.7-4.4 percentage points (*P* = .02) in Medicaid enrollment related to unemployment during 2020 compared with nonexpansion states, which had an increase of 0.2 (95% CI, –0.9 to 1.3) percentage points (*P* = .001) in partial year Medicaid enrollment.

### Sensitivity Analyses

As a supplement to the key analyses, we included specification checks to assess the robustness of the findings. In eTable 2 in the Supplement, we redefined unemployment such that unemployment occurring after March 2020 was the key source of exposure by linking the CPS-ASEC to monthly CPS data for 2020; this analysis was also reproduced by the Integrated Public Use Microdata Series team. This range included the peak unemployment period after the declaration of the COVID-19 pandemic as a public health emergency when unemployment reached 14.7% in April 2020, which was substantially higher than peak unemployment (9.9%) during the Great Recession of 2007 to 2008.^1,15^ Results obtained from this sensitivity analysis are comparable to the findings of the main analysis.

Analyses of aggregate changes in employment and coverage from 2019 to 2020 in expansion and nonexpansion states are included in eFigure 2, eFigure 3, and eTable 3 in the Supplement. From 2019 to 2020, unemployment increased by 6.3 (95% CI, 5.5-7.0) percentage points (*P* < .001) in nonexpansion states vs 8.2 (95% CI, 6.3-10.1) percentage points (*P* = .002) in expansion states. Similar differences in net changes in unemployment occurring between March and June 2020 in expansion and nonexpansion states were also observed (eTable 3 in the Supplement). Among adults with private coverage at baseline, the proportion with private coverage decreased by 11.6 (95% CI, −13.7 to −9.5) percentage points (*P* < .001), and the share with employer-sponsored health insurance decreased by 6.2 (95% CI, −9.0 to −3.5) percentage points (*P* < .001); these declines in insurance coverage were comparable in expansion and nonexpansion states. Nonexpansion states experienced a net increase of 1.8 (95% CI, 0.9-2.6) percentage points (*P* < .001) in Medicaid enrollment in 2020 compared with 2019 levels, but Medicaid expansion states experienced an increase of almost 3.3 (1.76 [95% CI, 0.92-2.60] + 1.58 [95% CI, 0.24-2.91] = 3.34) percentage points (*P* = .02) in enrollment. The proportion of uninsured adults increased by 5.8 percentage points (*P* < .001) in nonexpansion states but by 3.2 (5.75 [95% CI, 4.55-6.96) – 2.52 [95% CI, –3.85 to –1.18] = 3.23) percentage points (*P* < .01) in expansion states (eTable 3 in the Supplement). The findings in eTable 3 in the Supplement suggest that 51% of the newly unemployed workers with private coverage (and no previous Medicaid coverage) at baseline in nonexpansion states would have been able to enroll in Medicaid. Among the newly unemployed adults residing in expansion states, 65% would be able to enroll in Medicaid.

eTables 4 and 5 in the Supplement reapply this study’s empirical strategy to the 2018-to-2019 waves of the CPS-ASEC (calendar years 2017-2018). The proportion of workers in the sample who were unemployed during 2019 to 2020 was more than double the proportion that was unemployed during 2017 to 2018 (eTable 4 in the Supplement). The association between enrolling in Medicaid and unemployment was statistically significant (3.5 [95% CI, 1.0-6.1] percentage points; *P* < .001) for partial year enrollment rather than the entire year (1.8 [95% CI, −3.8-7.5] percentage points), and there was no detectable protection from unemployment-related coverage loss associated with state expansion status (eTable 5 in the Supplement). eTable 6 in the Supplement addresses additional potential concerns about biases arising from model specification issues.

## Discussion

The study’s findings provide new evidence on how Medicaid expansion moderated the association between incidental unemployment and coverage status. The onset of unemployment periods was a key factor in the loss of employer-sponsored health insurance coverage; however, net coverage loss owing to unemployment was lower in expansion states. Because of the job losses that largely occurred in 2020, newly unemployed workers could be expected to enroll in Medicaid to offset the loss of private coverage related to the COVID-19 crisis; however, this enrollment occurred more frequently in expansion states than in nonexpansion states. Medicaid enrollment was substantially higher in states with less restrictive Medicaid eligibility guidelines, and expanded Medicaid eligibility was a key component in improving the capacity for Medicaid enrollment among the unemployed.

At the onset of the COVID-19 pandemic (March 2020), the Coronavirus Aid, Relief, and Economic Security Act added enhanced funding for Medicaid-eligible and newly eligible services (ie, COVID-19 testing for uninsured individuals). However, the Act also created a provision to disregard unemployment insurance income in determining Medicaid eligibility based on the modified adjusted gross income formula.^16^ Future studies are needed to evaluate the association between this provision and Medicaid enrollment among unemployment insurance beneficiaries and, alternatively, unemployment insurance enrollment among these beneficiaries.

Although Medicaid is a vital component of the US health care system, the findings of the present study underscore Medicaid’s use as a component of the safety net—akin to how displaced workers could leverage unemployment insurance benefits to stabilize household financing.^17,18,19^ This study suggests that new Medicaid enrollment among newly unemployed workers was made more feasible with expanded Medicaid eligibility guidelines during the COVID-19 pandemic. In addition, the results support those of previous studies^5,20^ describing the use of broader eligibility guidelines for Medicaid to facilitate unemployment-related Medicaid enrollment during economic downturns (eg, the Great Recession from 2007 to 2009) and the increased accessibility to Medicaid among those affected by local economic downturns after passage of the ACA. In combination with other recent studies,^6,21^ we believe that this study’s findings provide support for potentially stabilizing access to care through Medicaid expansion for households affected by job loss.

Medicaid’s countercyclical nature remains an issue of policy interest. It was once understood and expected that Medicaid enrollment would increase as households experienced health insurance coverage loss associated with job instability during economic downturns, such as the ongoing downturn created by the COVID-19 pandemic.^22^ The volume of unemployment-related health insurance coverage losses was dependent on whether states had expanded Medicaid programs in place at the time of the downturn. In a related study examining the dynamics between employment, insurance coverage, and Medicaid expansion between 2014 and 2019, Benitez et al^20^ found that net coverage losses associated with declining economic conditions largely decreased in states after Medicaid expansion but not in nonexpansion states. In addition, the authors found that there was an increase in Medicaid enrollment associated with unemployment in expansion states compared with nonexpansion states.^20^ In another study of COVID-19–related Medicaid enrollment in North Carolina, a nonexpansion state, Shafer et al^23^ found unemployment-related enrollment to be more prevalent in communities with higher levels of prepandemic social vulnerability (eg, limited access to social services, lower levels of educational attainment, and housing insecurity). The risk of becoming uninsured after job loss was prevalent in both expansion and nonexpansion states.

### Limitations

This study has limitations. The COVID-19 crisis created issues of nonresponse bias hindering survey participation and could undermine data quality for the studies using the CPS-ASEC.^24,25,26^ Although the person-level fixed effects may account for unobservable yet time-invariant factors that could alter demand for maintaining coverage, some results could be attributable to workers having underlying, costly medical needs, making enrollment into Medicaid more valuable to maintaining their health and protecting them from the financial risk of health insurance coverage loss.^27^ Restricting the study’s attention to this focal population limited the sample size and may have limited the statistical power of the analyses. The smaller sample size may make it difficult to estimate the association between job loss and Medicaid enrollment across states more precisely.

There was limited capacity to assess the extent to which the new Medicaid enrollment was preceded by job loss. The CPS-ASEC asks about coverage and enrollment during the past year, and there is no indicator for (1) months without health insurance coverage of any kind or (2) months enrolled in Medicaid. The monthly CPS does not include survey items of coverage status—that feature is unique to the ASEC. Current coverage status is also captured in the ASEC, but data collection is largely limited to March of the survey year. Because of this limitation in what current coverage constitutes, the proportion of Medicaid enrollment occurring after the classification of COVID-19 as a public health emergency would be underestimated. For this reason, the present study examines changes in partial- and full-year Medicaid enrollment associated with unemployment. The findings show that unemployment-related Medicaid enrollment was largely associated with increases in partial-year enrollments, and this finding is consistent with people transitioning from private insurance coverage to Medicaid after experiencing job loss.

## Conclusions

In this cohort study of US adult workers, unemployment-related health insurance coverage loss was substantially higher in states that had yet to expand Medicaid, whereas expanded eligibility allowed Medicaid to potentially buffer households from periods without health insurance coverage owing to unanticipated job loss. The COVID-19 pandemic created unprecedented risks for becoming uninsured among working adults. Leveraging a longitudinal database allowed monitoring of transitions in health insurance coverage status and source among working adults before and during the pandemic.

## Notes

Supplement. **eMethods.** Framework for Regression Analysis **eTable 1.** Characteristics of the Study Sample **eTable 2.** COVID-Timed Unemployment and Associations With Changes in Coverage Among Workers in Medicaid Expansion and Nonexpansion States, 2019–2020 **eTable 3.** Changes in Employment and Coverage Status in Expansion and Nonexpansion States, 2019–2020 **eTable 4.** Unemployment-Associated Changes in Employment and Coverage Status in Expansion and Non-Expansion States, 2017-2018 **eTable 5.** Changes in Employment and Coverage Status in Expansion and Nonexpansion States, 2017–2018 **eTable 6.** Specification Tests of Regressions of Unemployment-Associated Changes in Medicaid Enrollment and Coverage Status in Expansion and Nonexpansion States, 2019–2020 **eFigure 1.** Sample Construction **eFigure 2.** 2019–2020 Changes in Employment Status Among Adults Employed in 2019 **eFigure 3.** 2019–2020 Changes in Coverage Status Among Adults Employed in 2019
