# Supplementary material for: Comparison of Unemployment-Related Health Insurance Coverage Changes in Medicaid Expansion vs Nonexpansion States During the COVID-19 Pandemic
Source: JAMA Health Forum. 2022 Jun 17;3(6):e221632. doi: 10.1001/jamahealthforum.2022.1632 (PMC9206185; doi:10.1001/jamahealthforum.2022.1632)
Supplement: Supplement. — eMethods. Framework for Regression Analysis eTable 1. Characteristics of the Study Sample eTable 2. COVID-Timed Unemployment and Associations With Changes in Coverage Among Workers in Medicaid Expansion and Nonexpansion States, 2019–2020 eTable 3. Changes in Employment and Coverage Status in Expansion and Nonexpansion States, 2019–2020 eTable 4. Unemployment-Associated Changes in Employment and Coverage Status in Expansion and Non-Expansion States, 2017-2018 eTable 5. Changes in Employment and Coverage Status in Expansion and Nonexpansion States, 2017–2018 eTable 6. Specification Tests of Regressions of Unemployment-Associated Changes in Medicaid Enrollment and Coverage Status in Expansion and Nonexpansion States, 2019–2020 eFigure 1. Sample Construction eFigure 2. 2019–2020 Changes in Employment Status Among Adults Employed in 2019 eFigure 3. 2019–2020 Changes in Coverage Status Among Adults Employed in 2019 [file jamahealthforum-e221632-s001.pdf]

Supplementary Online Content

Benitez J. Comparison of unemployment-related health insurance coverage changes in Medicaid expansion vs nonexpansion states during the COVID-19 pandemic. *JAMA Health Forum*. 3(6):e221632. doi:10.1001/jamahealthforum.2022.1632

eMethods. Framework for Regression Analysis
eTable 1. Characteristics of the Study Sample
eTable 2. COVID-Timed Unemployment and Associations With Changes in Coverage Among Workers in Medicaid Expansion and Nonexpansion States, 2019–2020
eTable 3. Changes in Employment and Coverage Status in Expansion and Nonexpansion States, 2019–2020
eTable 4. Unemployment-Associated Changes in Employment and Coverage Status in Expansion and Non-Expansion States, 2017-2018
eTable 5. Changes in Employment and Coverage Status in Expansion and Nonexpansion States, 2017–2018
eTable 6. Specification Tests of Regressions of Unemployment-Associated Changes in Medicaid Enrollment and Coverage Status in Expansion and Nonexpansion States, 2019–2020
eFigure 1. Sample Construction
eFigure 2. 2019–2020 Changes in Employment Status Among Adults Employed in 2019
eFigure 3. 2019–2020 Changes in Coverage Status Among Adults Employed in 2019

This supplementary material has been provided by the authors to give readers additional information about their work.

© 2022 Benitez J. *JAMA Health Forum*.

eFigure 1: Sample Construction

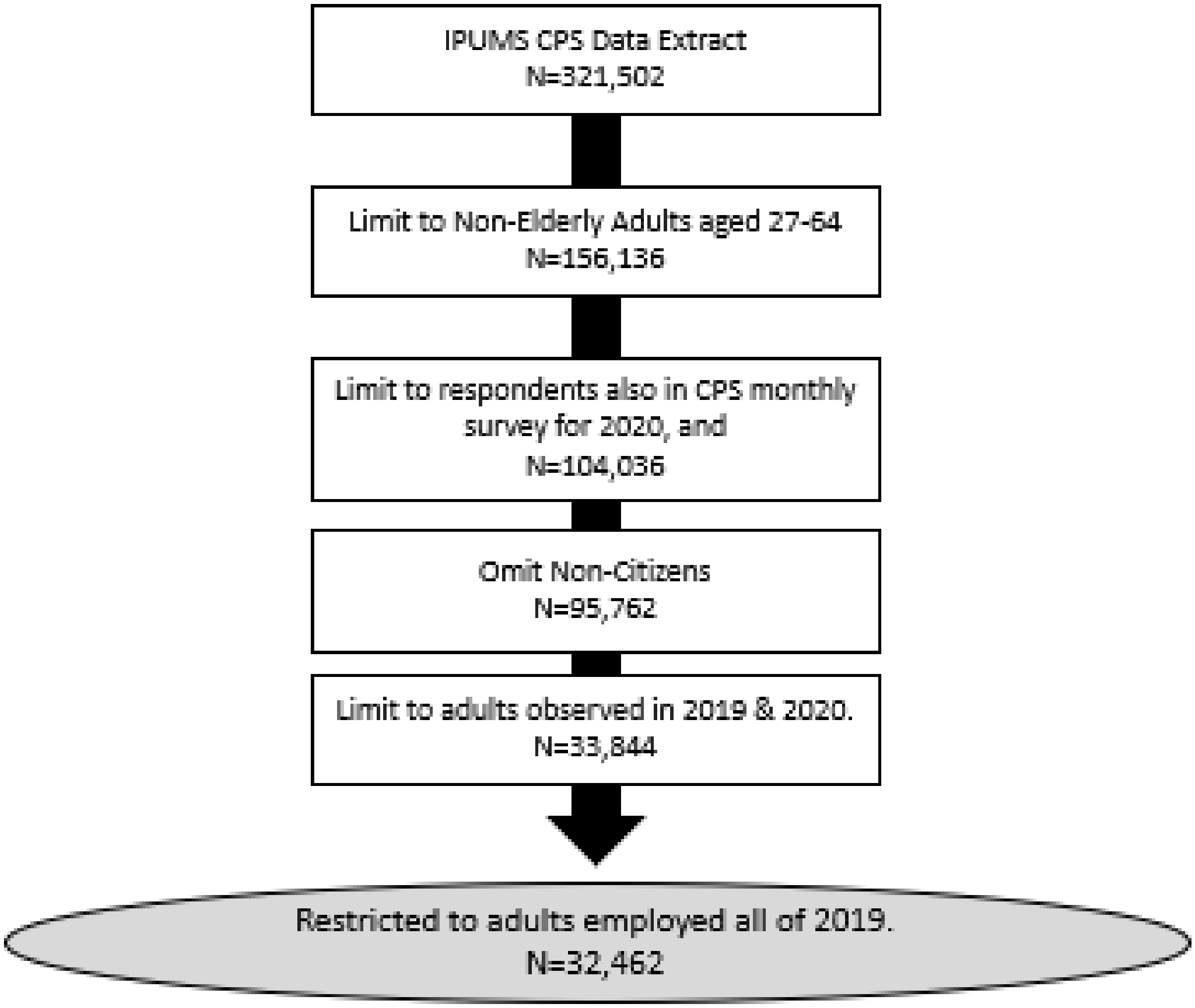

© 2022 Benitez J. *JAMA Health Forum*.

eMethods: Framework for Regression Analysis

$$Y_{ist} = \beta_0 + \beta_1 Unemployed_{it} + \beta_2(Unemployed_{it} \times Expansion_s) + \gamma X_{ist} + \mu_i + \delta_t + \varepsilon_{ist}$$

Our primary regression specification is outlined above, and the results from this approach are presented in Table 1 of the main text. Our approach compared differences in the impact of unemployment (i.e., becoming unemployed) during the COVID-19 pandemic in states with expanded Medicaid eligibility guidelines compared to states that did not expand Medicaid. Our key sources of variation were at the individual level (i.e., whether the sample person becomes unemployed) and policy variation at the state level (i.e., whether the state expanded Medicaid). Job losses occurring during between 2019 and 2020 could have been due to reasons aside from the shocks caused by the COVID-19 crisis. To allow our results to be more specific to the economic downturn caused by COVID, we restricted our sample to working aged (20-64) adults who were all employed at baseline.

Y_{ist} is the outcome associated with individual i in state s at time t. $Unemployed_{it}$ is a dummy variable that indicates whether sample person i had become unemployed in 2020. $Expansion_s$ is a dummy variable indicating if the sample person resided in a state that expanded Medicaid as of January 2020. β_1 provides the direction and strength of the association between becoming unemployed and the person's coverage status. For example, we anticipated becoming unemployed would be positively associated with reporting as uninsured (i.e., $\beta_1 > 0$). However, expanded access to Medicaid may have mitigated this relationship. If Medicaid expansion did facilitate more enrollments among those affected by job loss, then we expected residing in an expansion state to be protective from the full brunt of job loss. In which case, we expected that β_2, the coefficient on $Unemployed_{it} \times Expansion_s$, will be negative (i.e., $\beta_2 < 0$)—therefore, the net coverage losses due to job loss in expansion states would be attenuated. A main effect on $Expansion_s$ does not enter into the regression equation. As I estimate this model using a fixed effects framework, state expansion status would be collinear with the individual level fixed effect.

Our study is longitudinal with respect to observing a cohort of workers over two time points in 2019 and 2020. We included individual level fixed effects to control for important, yet time-invariant aspects unique to each sample person, μ_i. We also included a year fixed effects—δ_t—to capture changes in the levels of the outcome over time among workers that did not experience a job loss.

X_{ist} are time varying person-level controls like age and potentially time-varying controls like marital status, their unique industry/occupational code (which may also partially control for their risk of unemployment), and education. The individual-level fixed effects should absorb descriptors such as race, ethnicity, and sex (μ_i). However, small fractions (i.e., all <1%) of the sample indicated different race, ethnicities (e.g., Non-Hispanic in 2019 but Hispanic in 2020), and sex in their second year of the survey. Some of this could be due to random errors in data transcribing or even misclassification errors. With respect to sex, some of this could be due to limitations of large surveys such as the CPS or even the Behavioral Risk Factor Surveillance System in gender conforming and non-conforming respondents—or even how this may impact the validity demographic analyses of cross-sectional and even longitudinal analyses.[27]

© 2022 Benitez J. *JAMA Health Forum*.

eTable 1. Characteristics of the Study Sample

Variables	Medicaid Expansion States		Non-Expansion States		Difference
	Mean	(95% CI)	Mean	(95% CI)	
Age	46.7	(46.4, 46.9)	47.0	(46.6, 47.3)	-0.3*
Race/Ethnicity					
%Black/Non-Hispanic	6.9%	(6.4, 7.5)	12.4%	(11.5, 13.4)	-5.5***
%Hispanic (any race)	77.7%	(76.8, 78.6)	74.1%	(72.8, 75.4)	2.9***
%White/Non-Hispanic	8.4%	(7.8, 9)	9.1%	(8.3, 10)	3.3***
%Other Race/Non-Hispanic	7.0%	(6.5, 7.5)	4.4%	(3.8, 5.0)	-0.7*
Education					
%Less than high school diploma	5.0%	(4.6, 5.5)	6.4%	(5.7, 7.2)	-1.5***
%High school graduate	25.1%	(24.1, 26)	27.1%	(25.7, 28.4)	-1.9***
%Some college/technical school	25.8%	(24.8, 26.8)	28.8%	(27.4, 30.2)	-3.2***
%BA/BS+	44.1%	(43, 45.2)	37.7%	(36.2, 39.2)	6.6***
Other Demographic Characteristics					
%Female	51.2%	(50, 52.3)	52.5%	(51, 54)	-1.5**
%Male	48.8%	(47.7, 50)	47.5%	(46, 49)	1.5**
%Childless Adult	57.6%	(56.5, 58.6)	60.2%	(58.8, 61.7)	-2.1***
%Parent (1+ child in household)	42.4%	(41.4, 43.5)	39.8%	(38.3, 41.2)	2.1***
%Married	61.5%	(60.4, 62.7)	62.6%	(61.1, 64.1)	-0.7
%Metropolitan	87.2%	(86.4, 87.9)	83.5%	(82.4, 84.5)	3.9***
%Non-Metropolitan	12.8%	(12.1, 13.6)	16.5%	(15.5, 17.6)	-3.9***
Other Characteristics					
%Privately Insured at Baseline	79.1%	(78.2, 80)	76.1%	(74.8, 77.4)	2.8***
%Medicaid in 2019	13.0%	(12.2, 13.7)	8.5%	(7.6, 9.3)	4.7***
%Became Unemployed in 2020	8.2%	(7.6, 8.8)	6.3%	(5.6, 7.1)	1.9***
%Became Unemployed During March-April 2020 (Peak COVID)	7.3%	(6.7, 7.9)	5.2%	(4.6, 5.9)	2.2***
Sample size	10,751		5,502		

Source: Data are from the 2020-2021 IPUMS version of the Current Population Survey's Annual Social and Economic Supplement.

Note: * p<0.05, ** p<0.01, *** p<0.001. State Medicaid expansion was based on whether states had expanded Medicaid as of January 2020. Non-expansion states included: Alabama, Georgia, Florida, Kansas, Oklahoma, Missouri, Mississippi, North Carolina, Nebraska, South Carolina, South Dakota, Tennessee, Texas, Wisconsin, and Wyoming. Adults classified as Other/Non-Hispanics were 7.7% (n=1385) of the study sample. People of Asian descent (n=878; 4.8% of study sample) and American Indian/Alaska Natives (n=192; 1.1%) were the largest subgroups categorized as other. Additional subgroups included but were not limited to native Hawai'ians (n=70) and people identifying jointly as White/American Indian (n=89). All statistics presented are weighted to reflect the complex sampling design of the survey. All estimates in the table are rounded.

© 2022 Benitez J. *JAMA Health Forum*.

eTable 2. COVID-Timed Unemployment and their Associations with Changes in Coverage among Workers in Medicaid Expansion and Non-Expansion States, 2019-2020

A. Full Sample

	(1) Private (Any)	(2) ESI	(3) Marketplace	(4) Medicaid	(5) Medicaid Part of Year	(6) Medicaid All of Year	(7) Uninsured
Post-COVID Unemployment (March-June 2020)	-2.42	-0.83	1.32	3.55	1.11	2.44	-0.55
	[-9.86,5.03]	[-9.26,7.60]	[-1.83,4.47]	[0.87,6.24]	[-0.90,3.13]	[0.084,4.80]	[-7.81,6.72]
	(0.5175)	(0.8444)	(0.4037)	(0.0105)	(0.2724)	(0.0427)	(0.8800)
Expansion × Post-COVID Unemployment	1.79	-1.43	-1.23	-1.76	0.19	-1.95	0.71
	[-6.53,10.1]	[-10.3,7.44]	[-5.83,3.37]	[-5.86,2.33]	[-2.40,2.77]	[-5.33,1.43]	[-7.37,8.79]
	(0.6678)	(0.7471)	(0.5930)	(0.3917)	(0.8854)	(0.2530)	(0.8606)
N	32,462	32,462	32,462	32,462	32,462	32,462	32,462

B. Private Coverage

	(1) Private (Any)	(2) ESI	(3) Marketplace	(4) Medicaid	(5) Medicaid Part of Year	(6) Medicaid All of Year	(7) Uninsured
Post-COVID Unemployment (March-June 2020)	-5.82	-4.27	1.02	0.88	-0.097	0.97	6.43
	[-12.1,0.45]	[-14.2,5.64]	[-2.51,4.55]	[-2.14,3.89]	[-2.47,2.28]	[-2.20,4.15]	[-0.38,13.2]
	(0.0680)	(0.3911)	(0.5651)	(0.5620)	(0.9348)	(0.5413)	(0.0636)
Expansion × Post-COVID Unemployment	0.12	-2.54	-0.88	3.65	1.11	2.54	-3.05
	[-6.90,7.15]	[-12.8,7.67]	[-6.27,4.50]	[-0.54,7.83]	[-1.88,4.10]	[-1.29,6.37]	[-9.96,3.87]
	(0.9722)	(0.6191)	(0.7437)	(0.0864)	(0.4608)	(0.1892)	(0.3809)
N	25,086	25,086	25,086	25,086	25,086	25,086	25,086

C. Private Coverage with no Prior Medicaid

	(1) Private (Any)	(2) ESI	(3) Marketplace	(4) Medicaid	(5) Medicaid Part of Year	(6) Medicaid All of Year	(7) Uninsured
Post-COVID Unemployment (March-June 2020)	-6.42	-4.91	1.14	1.35	0.63	0.72	6.90
	[-13.1,0.30]	[-16.0,6.16]	[-2.50,4.78]	[-1.38,4.08]	[-0.85,2.10]	[-1.81,3.25]	[-0.21,14.0]
	(0.0608)	(0.3777)	(0.5326)	(0.3252)	(0.3968)	(0.5683)	(0.0569)
Expansion × Post-COVID Unemployment	1.05	-0.28	-1.40	2.91	0.10	2.81	-3.43
	[-6.32,8.42]	[-11.5,10.9]	[-6.91,4.11]	[-1.04,6.87]	[-1.79,2.00]	[-0.56,6.18]	[-10.7,3.79]
	(0.7754)	(0.9594)	(0.6111)	(0.1455)	(0.9151)	(0.0999)	(0.3447)
N	24,542	24,542	24,542	24,542	24,542	24,542	24,542

Source: Data are from the 2020-2021 IPUMS version of the Current Population Survey's Annual Social and Economic Supplement.

© 2022 Benitez J. *JAMA Health Forum*.

Notes: [*] $p < 0.05$, [**] $p < 0.01$, [***] $p < 0.001$. 95% confidence intervals in brackets. Results show coefficients from linear person-by-year fixed effects regression models. Coefficients are scaled by 100 for better interpretability. Regressions also include controls for marital status, education, sex, age, race/ethnicity, marital status, occupation/industry, and area-specific time trends. The sample is limited to adults US citizens aged 27-64. State Medicaid expansion was based on whether states had expanded as of January 2020.

© 2022 Benitez J. *JAMA Health Forum*.

eFigure 2. 2019-2020 Changes in Employment Status among Adults Employed in 2019

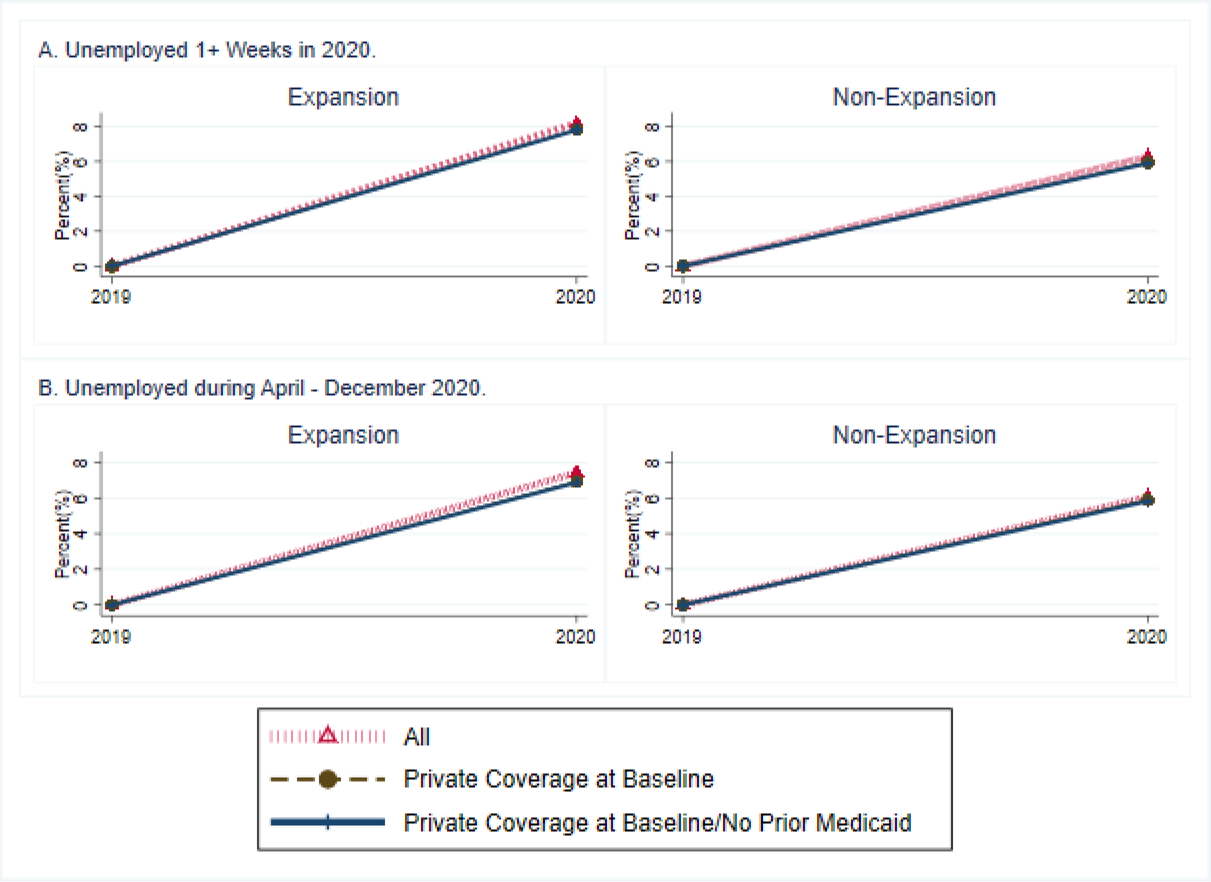

Source: Data are from the 2020-2021 IPUMS version of the Current Population Survey's Annual Social and Economic Supplement.

Notes: Samples above also limited to adults indicating they were not unemployed at any point in 2019--the base year for the analysis. This figure presents aggregate changes in in job loss based on whether they reported a) being unemployed for 1+ weeks during 2020 or b) a loss during March or April 2020. The sub-samples with prior coverage were sample persons having either ESI or marketplace coverage in 2019. Sub-samples with prior private coverage and no prior Medicaid were not enrolled in Medicaid in 2019. States that had not expanded Medicaid as of January 2020 were classified as non-expansion states: AL, GA, FL, KS, OK, MO, MS, NC, NE, SC, SD, TN, TX, WI, and WY. All statistics presented are weighted to reflect the complex sampling design of the survey.

© 2022 Benitez J. *JAMA Health Forum*.

eFigure 3. 2019-2020 Changes in Coverage Status among Adults Employed in 2019

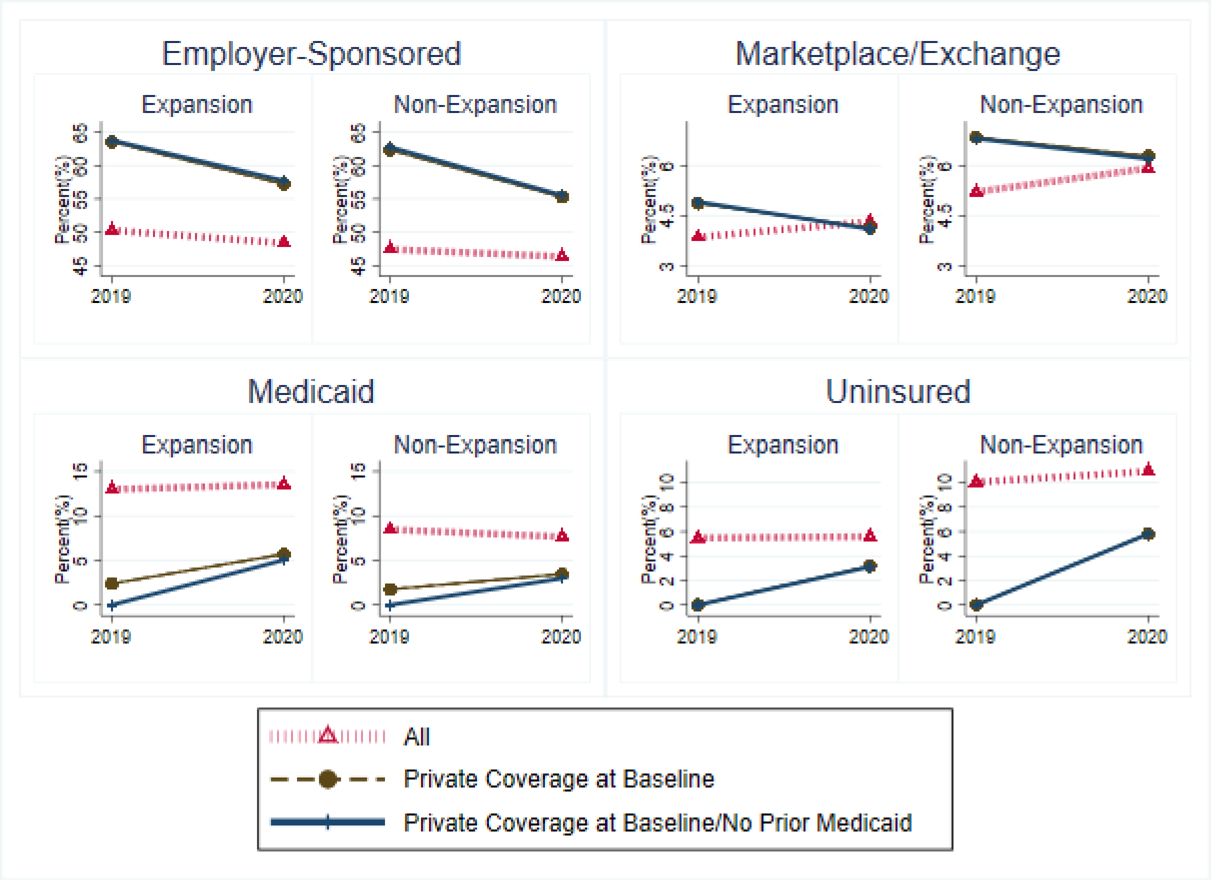

Source: Data are from the 2020-2021 IPUMS version of the Current Population Survey's Annual Social and Economic Supplement.

Notes: Samples above also limited to adults indicating they were not unemployed at any point in 2019--the base year for the analysis. This figure presents aggregate changes in coverage rather than those that may be attributable to job loss. The sub-samples with prior coverage were sample persons having either ESI or marketplace coverage in 2019. Sub-samples with prior private coverage and no prior Medicaid were not enrolled in Medicaid in 2019. States that had not expanded Medicaid as of January 2020 were classified as non-expansion states: AL, GA, FL, KS, OK, MO, MS, NC, NE, SC, SD, TN, TX, WI, and WY. All statistics presented are weighted to reflect the complex sampling design of the survey.

© 2022 Benitez J. *JAMA Health Forum*.

eTable 3. Changes in Employment and Coverage Status in Expansion and Non-Expansion States, 2019-2020

$$Y_{ist} = \beta_0 + \beta_1 Y2020_t + \beta_2(Expansion_s \times Y2020_t) + \gamma X_{ist} + \mu_i + \delta_t + \varepsilon_{ist}$$

My secondary analysis used a more standard difference-in-differences approach. We still leveraged the longitudinal aspect of the CPS-ASEC, and we included person-level fixed effects to account for unobserved heterogeneity across individuals that may be correlated with the outcomes. The only new variable compared to that presented in the main text is $Y2020_t$—a dummy variable to indicate capture the changes in levels of the outcome over the two-year period (2019-2020) for workers in non-expansion states. β_2, the coefficient on $Expansion_s \times Y2020_t$, captures the relative differences in the changes of the outcomes among expansion workers in expansion states relative to workers in non-expansion over the timespan. This secondary approach does not rely on the onset of unemployment like the main analyses—rather we focus on the changes in levels of outcomes that are a function of time—time mostly being the onset of the 2020 COVID-19 crisis.

A. Full Sample

	(1) Unemployed	(2) Unemployed After COVID	(3) Private (Any)	(4) ESI	(5) Marketplace	(6) Medicaid	(7) Medicaid Part of Year	(8) Medicaid All of Year	(9) Uninsured
Post	6.26	6.21	-0.39	-0.34	0.60	-0.76	-0.46	-0.30	0.61
	[5.52,6.99]	[5.60,6.83]	[-2.30,1.52]	[-2.03,1.36]	[-0.44,1.64]	[-1.97,0.46]	[-0.77,-0.15]	[-1.29,0.70]	[-0.54,1.75]
	(0.0000)	(0.0000)	(0.6850)	(0.6925)	(0.2500)	(0.2190)	(0.0048)	(0.5506)	(0.2937)
Expansion × Year 2020	1.92	1.64	0.13	-0.80	-0.31	0.97	0.25	0.72	-0.63
	[0.76,3.08]	[0.44,2.84]	[-2.01,2.27]	[-2.79,1.20]	[-1.56,0.95]	[-0.32,2.26]	[-0.25,0.75]	[-0.37,1.82]	[-2.07,0.81]
	(0.0017)	(0.0083)	(0.9019)	(0.4272)	(0.6250)	(0.1375)	(0.3243)	(0.1920)	(0.3872)
2019 Mean for Non-Expansion States	0	0	76.1	47.4	5.2	8.5	1	7.5	10
2019 Mean for Expansion States	0	0	79.1	50.3	3.9	13	1.4	11.6	5.5
Observations	32,506	32,506	32,506	32,506	32,506	32,506	32,506	32,506	32,506

B. Private Coverage in 2019

	(1) Unemployed	(2) Unemployed After COVID	(3) Private (Any)	(4) ESI	(5) Marketplace	(6) Medicaid	(7) Medicaid Part of Year	(8) Medicaid All of Year	(9) Uninsured
	b/ci95/p	b/ci95/p	b/ci95/p	b/ci95/p	b/ci95/p	b/ci95/p	b/ci95/p	b/ci95/p	b/ci95/p
Post	5.85	5.97	-11.6	-6.24	-0.75	1.76	-0.33	2.09	5.75
	[4.89,6.80]	[5.27,6.66]	[-13.7,-9.50]	[-9.00,-3.48]	[-1.98,0.49]	[0.92,2.60]	[-0.76,0.094]	[1.29,2.89]	[4.55,6.96]
	(0.0000)	(0.0000)	(0.0000)	(0.0000)	(0.2311)	(0.0001)	(0.1239)	(0.0000)	(0.0000)
Expansion × Year 2020	1.85	1.35	2.50	0.48	-0.25	1.58	0.20	1.37	-2.52
	[0.50,3.21]	[0.13,2.58]	[0.24,4.76]	[-2.45,3.42]	[-1.76,1.27]	[0.24,2.91]	[-0.35,0.76]	[0.28,2.46]	[-3.85,-1.18]
	(0.0084)	(0.0313)	(0.0311)	(0.7432)	(0.7456)	(0.0216)	(0.4683)	(0.0145)	(0.0004)
2019 Mean for Non-Expansion States	0	0	100	62.3	6.80	1.7	.600	1.2	0

© 2022 Benitez J. *JAMA Health Forum*.

2019 Mean for Expansion States	0	0	100	63.5	4.9	2.4	1	1.4	0
Observations	25,154	25,154	25,154	25,154	25,154	25,154	25,154	25,154	25,154

C. Private Coverage in 2019/No Prior Medicaid

	(1) Unemployed	(2) Unemployed After COVID	(3) Private (Any)	(4) ESI	(5) Marketplace	(6) Medicaid	(7) Medicaid Part of Year	(8) Medicaid All of Year	(9) Uninsured
	b/ci95/p	b/ci95/p	b/ci95/p	b/ci95/p	b/ci95/p	b/ci95/p	b/ci95/p	b/ci95/p	b/ci95/p
Post	5.81	5.87	-11.2	-6.40	-0.90	2.95	0.29	2.66	5.75
	[4.85,6.77]	[5.14,6.60]	[-13.3,-9.11]	[-9.11,-3.70]	[-2.30,0.49]	[1.94,3.96]	[0.062,0.52]	[1.77,3.55]	[4.52,6.98]
	(0.0000)	(0.0000)	(0.0000)	(0.0000)	(0.1996)	(0.0000)	(0.0139)	(0.0000)	(0.0000)
Expansion × Year 2020	1.87	1.37	2.63	0.90	-0.30	2.03	0.55	1.49	-2.56
	[0.55,3.18]	[0.086,2.64]	[0.38,4.88]	[-1.93,3.72]	[-1.93,1.32]	[0.63,3.43]	[0.19,0.90]	[0.32,2.66]	[-3.92,-1.20]
	(0.0065)	(0.0369)	(0.0227)	(0.5270)	(0.7074)	(0.0053)	(0.0033)	(0.0138)	(0.0004)
2019 Mean for Non-Expansion States	0	0	100	62.7	6.80	0	0	0	0
2019 Mean for Expansion States	0	0	100	63.8	4.9	0	0	0	0
Observations	24,616	24,616	24,616	24,616	24,616	24,616	24,616	24,616	24,616

Source: Data are from the 2020-2021 IPUMS version of the Current Population Survey's Annual Social and Economic Supplement.

Notes: * $p < 0.05$, ** $p < 0.01$, *** $p < 0.001$. 95% confidence intervals in brackets. Results show coefficients from linear person-by-year fixed effects regression models. Coefficients are scaled by 100 for better interpretability. Regressions also include controls for marital status, education, sex, age, race/ethnicity, marital status, occupation/industry, and area-specific time trends. The sample is limited to adults US citizens aged 27-64. Samples above also limited to adults indicating they were not unemployed at any point in 2019--the base year for the analysis. State Medicaid expansion was based on whether states had expanded Medicaid as of January 2020. All statistics presented are weighted to reflect the complex sampling design of the survey.

© 2022 Benitez J. *JAMA Health Forum*.

eTable 4. Unemployment-Associated Changes in Employment and Coverage Status in Expansion and Non-Expansion States, 2017-2018

A. Full Sample

	(1) Private (Any)	(2) ESI	(3) Marketplace	(4) Medicaid	(5) Medicaid Part of Year	(6) Medicaid All of Year	(7) Uninsured
Unemployment	-7.27	3.48	2.04	1.68	0.066	3.15	3.28
	[-13.7,-0.89]	[-5.35,12.3]	[-1.45,5.53]	[-3.88,7.24]	[-1.09,1.22]	[-4.44,10.7]	[-1.87,8.43]
	(0.0264)	(0.4328)	(0.2452)	(0.5461)	(0.9092)	(0.4090)	(0.2072)
Expansion × Unemployment	2.84	-9.90	-1.19	1.18	5.40	0.36	1.13
	[-5.26,10.9]	[-20.7,0.87]	[-6.00,3.62]	[-5.72,8.09]	[2.56,8.23]	[-8.02,8.74]	[-5.85,8.11]
	(0.4851)	(0.0708)	(0.6217)	(0.7319)	(0.0004)	(0.9317)	(0.7468)
N	36,180	36,180	36,180	36,180	36,180	36,180	36,180

B. Private Coverage at Baseline

	(1) Private (Any) b/ci95/p	(2) ESI b/ci95/p	(3) Marketplace b/ci95/p	(4) Medicaid b/ci95/p	(5) Medicaid Part of Year b/ci95/p	(6) Medicaid All of Year b/ci95/p	(7) Uninsured b/ci95/p
Unemployment	-11.2	4.26	2.11	-0.82	0.15	2.77	7.82
	[-17.4,-4.91]	[-7.14,15.7]	[-3.69,7.92]	[-6.78,5.14]	[-1.06,1.36]	[-2.28,7.81]	[-1.08,16.7]
	(0.0008)	(0.4563)	(0.4682)	(0.7834)	(0.8078)	(0.2757)	(0.0836)
Expansion × Unemployment	3.28	-13.6	-0.099	5.88	3.50	0.40	-2.84
	[-4.45,11.0]	[-28.1,0.80]	[-7.63,7.43]	[-2.79,14.5]	[1.09,5.92]	[-5.45,6.25]	[-13.9,8.23]
	(0.3982)	(0.0635)	(0.9792)	(0.1795)	(0.0054)	(0.8912)	(0.6089)
N	27,982	27,982	27,982	27,982	27,982	27,982	27,982

C. Private Coverage at Baseline with no prior Medicaid

	(1) Private (Any) b/ci95/p	(2) ESI b/ci95/p	(3) Marketplace b/ci95/p	(4) Medicaid b/ci95/p	(5) Medicaid Part of Year b/ci95/p	(6) Medicaid All of Year b/ci95/p	(7) Uninsured b/ci95/p
Unemployment	-9.78	5.97	1.66	2.28	0.12	2.16	7.77
	[-14.9,-4.66]	[-4.56,16.5]	[-3.01,6.33]	[-3.02,7.58]	[-1.14,1.38]	[-2.79,7.12]	[-0.80,16.3]
	(0.0004)	(0.2604)	(0.4791)	(0.3921)	(0.8538)	(0.3847)	(0.0747)
Expansion × Unemployment	1.86	-16.5	0.81	5.35	3.54	1.81	-3.82
	[-4.82,8.54]	[-30.2,-2.76]	[-6.09,7.71]	[-0.83,11.5]	[1.02,6.05]	[-3.84,7.47]	[-15.3,7.70]
	(0.5790)	(0.0196)	(0.8148)	(0.0882)	(0.0068)	(0.5229)	(0.5084)
N	26,822	26,822	26,822	26,822	26,822	26,822	26,822

© 2022 Benitez J. *JAMA Health Forum*.

Source: Data are from the 2018-2019 IPUMS version of the Current Population Survey's Annual Social and Economic Supplement.
Notes: * $p < 0.05$, ** $p < 0.01$, *** $p < 0.001$. 95% confidence intervals in brackets. Results show coefficients from linear person-by-year fixed effects regression models. Coefficients are scaled by 100 for better interpretability. Regressions also include controls for marital status, education, sex, age, race/ethnicity, marital status, occupation/industry, and area-specific time trends. The sample is limited to adults US citizens aged 27-64. Samples above also limited to adults indicating they were not unemployed at any point in 2017--the base year for the analysis. State Medicaid expansion was based on whether states had expanded Medicaid as of 2016. All statistics presented are weighted to reflect the complex sampling design of the survey.

© 2022 Benitez J. *JAMA Health Forum*.

eTable 5. Changes in Employment and Coverage Status in Expansion and Non-Expansion States, 2017-2018.

A. Full Sample

	(1) Unemployed b/ci95/p	(2) Private (Any) b/ci95/p	(3) ESI b/ci95/p	(4) Marketplace b/ci95/p	(5) Medicaid b/ci95/p	(6) Medicaid Part of Year b/ci95/p	(7) Medicaid All of Year b/ci95/p	(8) Uninsured b/ci95/p
Year 2018	2.98	1.14	0.66	5.51	-0.99	0.59	7.15	-2.58
	[2.48,3.48]	[-0.086,2.38]	[-0.66,1.98]	[4.38,6.64]	[-1.81,-0.16]	[0.31,0.87]	[6.10,8.20]	[-3.69,-1.47]
	(0.0000)	(0.0677)	(0.3195)	(0.0000)	(0.0203)	(0.0001)	(0.0000)	(0.0000)
Expansion × 2018	0.14	-0.79	0.39	-1.49	-0.74	0.69	4.18	1.27
	[-0.55,0.83]	[-2.61,1.03]	[-1.22,2.00]	[-2.90,-0.086]	[-1.98,0.50]	[0.30,1.08]	[2.22,6.14]	[-0.082,2.62]
	(0.6771)	(0.3881)	(0.6303)	(0.0380)	(0.2371)	(0.0008)	(0.0001)	(0.0650)
2017 Mean for Non-Expansion States	0	74.8	44.5	0	8.5	0	0	13.4
2017 Mean for Expansion States	0	79.7	48.7	0	14.3	0	0	6.7
Observations	36,214	36,214	36,214	36,214	36,214	36,214	36,214	36,214

B. Private Coverage at Baseline

	(1) Unemployed	(2) Private (Any)	(3) ESI	(4) Marketplace	(5) Medicaid	(6) Medicaid Part of Year	(7) Medicaid All of Year	(8) Uninsured
Year 2018	2.65	-11.3	-5.49	5.63	0.46	0.28	3.03	4.14
	[2.12,3.19]	[-12.6,-10.0]	[-7.27,-3.71]	[4.49,6.77]	[-0.46,1.38]	[0.073,0.49]	[2.15,3.92]	[2.87,5.41]
	(0.0000)	(0.0000)	(0.0000)	(0.0000)	(0.3197)	(0.0090)	(0.0000)	(0.0000)
Expansion × 2018	0.20	1.76	1.52	-1.52	0.48	0.45	1.97	-1.38
	[-0.48,0.87]	[-0.41,3.92]	[-0.29,3.33]	[-2.96,-0.072]	[-0.66,1.63]	[0.15,0.74]	[0.52,3.42]	[-2.79,0.021]
	(0.5607)	(0.1094)	(0.0977)	(0.0400)	(0.4030)	(0.0039)	(0.0086)	(0.0533)
2017 Mean for Non-Expansion States	0	100	59.5	0	2.8	0	0	2.2
2017 Mean for Expansion States	0	100	61.1	0	4.80	0	0	1
Observations	28,036	28,036	28,036	28,036	28,036	28,036	28,036	28,036

C. Private Coverage at Baseline with no prior Medicaid

	(1) Unemployed	(2) Private (Any)	(3) ESI	(4) Marketplace	(5) Medicaid	(6) Medicaid Part of Year	(7) Medicaid All of Year	(8) Uninsured
Year 2018	2.58	-10.9	-5.35	5.58	2.77	0.29	2.48	4.08
	[2.03,3.13]	[-12.2,-9.62]	[-7.17,-3.52]	[4.47,6.69]	[2.12,3.42]	[0.075,0.50]	[1.70,3.26]	[2.84,5.32]
	(0.0000)	(0.0000)	(0.0000)	(0.0000)	(0.0000)	(0.0090)	(0.0000)	(0.0000)
Expansion × 2018	0.24	2.29	1.75	-1.57	2.09	0.39	1.70	-1.51
	[-0.49,0.97]	[0.43,4.15]	[-0.079,3.57]	[-3.00,-0.15]	[0.88,3.30]	[0.10,0.68]	[0.49,2.91]	[-2.89,-0.13]

© 2022 Benitez J. *JAMA Health Forum*.

	(0.5166)	(0.0168)	(0.0604)	(0.0309)	(0.0011)	(0.0087)	(0.0069)	(0.0330)
2017 Mean for Non-Expansion States	0	100	59.9	0	0	0	0	2.2
2017 Mean for Expansion States	0	100	61.7	0	0	0	0	1.1
Observations	26,874	26,874	26,874	26,874	26,874	26,874	26,874	26,874

Source: Data are from the 2018-2019 IPUMS version of the Current Population Survey's Annual Social and Economic Supplement.
Notes: * $p < 0.05$, ** $p < 0.01$, *** $p < 0.001$. 95% confidence intervals in brackets. Results show coefficients from linear person-by-year fixed effects regression models. Coefficients are scaled by 100 for better interpretability. Regressions also include controls for marital status, education, sex, age, race/ethnicity, marital status, occupation/industry, and area-specific time trends. The sample is limited to adults US citizens aged 27-64. Samples above also limited to adults indicating they were not unemployed at any point in 2017--the base year for the analysis. State Medicaid expansion was based on whether states had expanded Medicaid as of 2016. All statistics presented are weighted to reflect the complex sampling design of the survey. In the table above, we use the same model specification used in eTable 5. However, the sample is limited to a panel of workers observed in both 2017 and 2018. In terms of magnitude, the share of workers that would become unemployed between 2019 and 2020 was more than double the share that would become unemployed during 2017-2018.

© 2022 Benitez J. *JAMA Health Forum*.

eTable 6. Specification Tests of Regressions of Unemployment-Associated Changes in Medicaid Enrollment and Coverage Status in Expansion and Non-Expansion States, 2019-2020

The following regression results serve as specification tests comparing the results of our preferred approach using individual fixed effects (all in column 4) against alternative model specifications using nonlinear binomial fixed effects logit models (1) and linear model specifications in (2) and (3). Survey weights were used in models (1), (2), and (4), but only model (4) incorporated individual fixed effects. Models (1) – (3) allow for detection of baseline differences across state expansion status, but those presented are non-substantive differences. The specification in (4) is preferred because it minimizes the threat of omitted variable bias arising from important, yet unobservable differences across individuals that are time invariant and could explain their propensity to become unemployed as well as their propensity to have a change in their coverage status. Model (4) is also able to utilize all of the available observations while the logit model presented in (1) drop an overwhelming share of the study observations. This problem arises in nonlinear models where observations' characteristics perfectly predict the outcome—in this case, it was found that the individual fixed effects perfectly predicted much of the variation in the outcomes. This is why it was important to account for them in (4) although this comes as a tradeoff. The objective of this was not to produce predicted values of coverage across each state corresponding to whether workers became unemployed—rather this study was to explore magnitudes of the association (i.e., the coefficients) between job loss and coverage changes across states that did and did not expand Medicaid under the Affordable Care Act.

eTable 6. Specification Tests of Regressions of Unemployment-Associated Changes in Medicaid Enrollment and Coverage Status in Expansion and Non-Expansion States, 2019-2020

Panel A. Medicaid

	(1)<br>Logit<br>b/ci95/p	(2)<br>OLS<br>b/ci95/p	(3)<br>Random Effects<br>b/ci95/p	(4)<br>Fixed Effects<br>b/ci95/p
1.mcdexpand2020	0.86	1.20	1.27	
	[0.054,1.67]	[-0.10,2.50]	[0.18,2.37]	
	(0.0365)	(0.0706)	(0.0230)	
1.unempl	0.25	0.87	1.31	0.72
	[-0.44,0.95]	[-1.28,3.03]	[-0.76,3.38]	[-1.88,3.32]
	(0.4738)	(0.4188)	(0.2159)	(0.5806)
1.mcdexpand2020#1.unempl	0.64	4.64	5.82	4.42
	[-0.14,1.41]	[1.27,8.00]	[2.24,9.40]	[0.57,8.28]
	(0.1064)	(0.0080)	(0.0015)	(0.0252)
2020.year			-0.29	
			[-0.71,0.12]	
			(0.1621)	

© 2022 Benitez J. *JAMA Health Forum*.

Observations	8,097	24,574	24,616	24,542
R-Square		0.100		0.546
Area-Specific Time Trend	Yes	Yes	Yes	Yes
Individual Fixed Effect	No	No	No	Yes
Survey Weights Used	Yes	Yes	No	Yes

Panel B. Medicaid, Part of Year

	(1)<br>Logit<br>b/ci95/p	(2)<br>OLS<br>b/ci95/p	(3)<br>Random Effects<br>b/ci95/p	(4)<br>Fixed Effects<br>b/ci95/p
1.mcdexpand2020	1.92	0.57	0.64	
	[0.32,3.52]	[0.067,1.07]	[0.16,1.11]	
	(0.0184)	(0.0270)	(0.0088)	
1.unempl	2.03	0.55	0.92	0.18
	[0.63,3.43]	[-0.36,1.46]	[-0.27,2.11]	[-0.88,1.25]
	(0.0045)	(0.2286)	(0.1309)	(0.7301)
1.mcdexpand2020#1.unempl	0.038	2.56	2.55	2.88
	[-1.49,1.56]	[1.00,4.13]	[0.75,4.35]	[1.14,4.62]
	(0.9612)	(0.0018)	(0.0055)	(0.0016)
2020.year			-0.16	
			[-0.39,0.074]	
			(0.1810)	
Observations	2,920	24,574	24,616	24,542
R-Square		0.056		0.537
Area-Specific Time Trend	Yes	Yes	Yes	Yes
Individual Fixed Effect	No	No	No	Yes
Survey Weights Used	Yes	Yes	No	Yes

Source: Data are from the 2020-2021 IPUMS version of the Current Population Survey's Annual Social and Economic Supplement.
Notes: * $p < 0.05$, ** $p < 0.01$, *** $p < 0.001$. 95% confidence intervals in brackets. Results show coefficients from linear person-by-year fixed effects regression models. Coefficients from linear regression models (2)-(4) are scaled by 100 for better interpretability. Regressions also include controls for marital status, education, sex, age, race/ethnicity, marital status, and occupation/industry. The sample is limited to adults US citizens aged 27-64. Samples above also limited to adults indicating they were not unemployed at any point in 2019, had private coverage in 2019, as well as had not been enrolled in Medicaid during 2019. Medicaid expansion was based on whether states had expanded Medicaid as of January 2020.

© 2022 Benitez J. *JAMA Health Forum*.

eTable 6. Specification Tests of Regressions of Unemployment-Associated Changes in Medicaid Enrollment and Coverage Status in Expansion and Non-Expansion States, 2019-2020 (continued).

Panel C. Medicaid, All Year

	(1)<br>Logit<br>b/ci95/p	(2)<br>OLS<br>b/ci95/p	(3)<br>Random Effects<br>b/ci95/p	(4)<br>Fixed Effects<br>b/ci95/p
1.mcdexpand2020	0.59	0.63	0.64	
	[-0.21,1.39]	[-0.42,1.68]	[-0.28,1.55]	
	(0.1480)	(0.2360)	(0.1731)	
1.unempl	-0.13	0.32	0.39	0.54
	[-0.98,0.73]	[-1.54,2.19]	[-1.58,2.36]	[-1.68,2.75]
	(0.7727)	(0.7308)	(0.6988)	(0.6303)
1.mcdexpand2020#1.unempl	0.53	2.07	3.27	1.54
	[-0.43,1.50]	[-0.95,5.09]	[0.18,6.35]	[-1.63,4.72]
	(0.2796)	(0.1740)	(0.0380)	(0.3339)
2020.year			-0.14	
			[-0.55,0.27]	
			(0.5162)	
Observations	7,672	24,574	24,616	24,542
R-Square		0.091		0.543
Area-Specific Time Trend	Yes	Yes	Yes	Yes
Individual Fixed Effect	No	No	No	Yes
Survey Weights Used	Yes	Yes	No	Yes

Panel D. Uninsured

	(1)<br>Logit<br>b/ci95/p	(2)<br>OLS<br>b/ci95/p	(3)<br>Random Effects<br>b/ci95/p	(4)<br>Fixed Effects<br>b/ci95/p
1.mcdexpand2020	-0.26	-0.56	-0.27	
	[-1.08,0.56]	[-2.36,1.24]	[-1.58,1.04]	
	(0.5328)	(0.5339)	(0.6894)	
1.unempl	1.48	10.9	10.9	11.1
	[0.87,2.09]	[3.80,18.1]	[4.85,17.0]	[2.62,19.5]
	(0.0000)	(0.0034)	(0.0004)	(0.0113)
1.mcdexpand2020#1.unempl	-1.08	-8.91	-7.79	-8.40
	[-1.83,-0.32]	[-16.2,-1.64]	[-14.1,-1.43]	[-16.8,-0.0047]
	(0.0050)	(0.0173)	(0.0163)	(0.0499)
2020.year			-1.40	
			[-2.07,-0.72]	

© 2022 Benitez J. *JAMA Health Forum*.

			(0.0000)	
Observations	7,821	24,574	24,616	24,542
R-Square		0.109		0.554
Area-Specific Time Trend	Yes	Yes	Yes	Yes
Individual Fixed Effect	No	No	No	Yes
Survey Weights Used	Yes	Yes	No	Yes

Source: Data are from the 2020-2021 IPUMS version of the Current Population Survey's Annual Social and Economic Supplement.
Notes: * p < 0.05, ** p < 0.01, *** p < 0.001. 95% confidence intervals in brackets. Results show coefficients from linear person-by-year fixed effects regression models. Coefficients from linear regression models (2)-(4) are scaled by 100 for better interpretability. Regressions also include controls for marital status, education, sex, age, race/ethnicity, marital status, and occupation/industry. The sample is limited to adults US citizens aged 27-64. Samples above also limited to adults indicating they were not unemployed at any point in 2019, had private coverage in 2019, as well as had not been enrolled in Medicaid during 2019. Medicaid expansion was based on whether states had expanded Medicaid as of January 2020.

© 2022 Benitez J. *JAMA Health Forum*.
